# Supplementary material for: Factors associated with and socioeconomic inequalities in underweight, overweight and obesity among adults aged 18–49 years in Lesotho: Evidence from the 2023–2024 Demographic and Health Survey
Source: PLOS Glob Public Health. 2026 Jan 20;6(1):e0005555. doi: 10.1371/journal.pgph.0005555 (PMC12818733; doi:10.1371/journal.pgph.0005555)
Supplement: S1 Table — (DOCX) [file pgph.0005555.s001.docx]

**S1 Table: Prevalence of underweight, normal weight, and overweight/obesity by socio-demographic characteristics among male participants aged 18–49 years, LDHS 2023–2024**

| **Variables** | **BMI (%)** | | | **Pearson χ² (df)** | **Design-based F (df1, df2)** | ***P*-value*** |
| --- | --- | --- | --- | --- | --- | --- |
|  | **Underweight** | **Normal BMI** | **Overweight/Obesity** |  |  |  |
| **Age Group** |  |  |  | χ²(4)=244.20 | F(3.89,1442.92)=14.86 | <0.001 |
| 18–29 | 22.43 | 69.63 | 7.94 |  |  |  |
| 30–39 | 23.74 | 55.29 | 20.98 |  |  |  |
| 40–49 | 21.7 | 53.48 | 24.83 |  |  |  |
| **Education** |  |  |  | χ²(4)=198.51 | F(3.71,1377.26)=11.38 | <0.001 |
| No education or primary | 22.59 | 66.25 | 11.15 |  |  |  |
| Secondary | 22.32 | 62.34 | 15.33 |  |  |  |
| Higher | 23.58 | 45.5 | 30.92 |  |  |  |
| **Marital Status** |  |  |  | χ²(4)=247.98 | F(3.80,1409.84)=17.63 | <0.001 |
| Never married | 25.23 | 66.65 | 8.12 |  |  |  |
| Married | 19.63 | 55.77 | 24.59 |  |  |  |
| Widowed/Divorce/Separated | 23.69 | 60.89 | 15.41 |  |  |  |
| **Wealth Index** |  |  |  | χ²(8)=413.86 | F(7.15,2653.18)=15.34 | <0.001 |
| Poorest | 19.35 | 74.42 | 6.23 |  |  |  |
| Poorer | 26.17 | 68.12 | 5.71 |  |  |  |
| Middle | 22.77 | 65.61 | 11.62 |  |  |  |
| Richer | 24.19 | 54.13 | 21.68 |  |  |  |
| Richest | 20.16 | 48.27 | 31.57 |  |  |  |
| **Ecological Zone** |  |  |  | χ²(6)=141.85 | F(4.74,1759.40)=10.02 | <0.001 |
| Lowlands | 24.96 | 56.92 | 18.12 |  |  |  |
| Foothills | 26 | 67.63 | 6.37 |  |  |  |
| Mountains | 12.92 | 74.53 | 12.56 |  |  |  |
| Senqu River Valley | 15.56 | 72.27 | 12.16 |  |  |  |
| **Region of Residence** |  |  |  | χ²(18)=177.92 | F(12.13,4499.57)=4.32 | <0.001 |
| Butha-Buthe | 19.99 | 66.03 | 13.98 |  |  |  |
| Leribe | 23.49 | 64.59 | 11.92 |  |  |  |
| Berea | 21.15 | 56.98 | 21.86 |  |  |  |
| Maseru | 28.77 | 53.32 | 17.92 |  |  |  |
| Mafeteng | 21.07 | 63.8 | 15.13 |  |  |  |
| Mohale's Hoek | 17.46 | 69.86 | 12.69 |  |  |  |
| Quthing | 17.78 | 68.64 | 13.57 |  |  |  |
| Qacha's Nek | 15.52 | 68.38 | 16.11 |  |  |  |
| Mokhotlong | 18.86 | 67 | 14.13 |  |  |  |
| Thaba-Tseka | 5.69 | 82.04 | 12.27 |  |  |  |
| **Place of Residence** |  |  |  | χ²(2)=179.30 | F(1.94,718.69)=18.33 | <0.001 |
| Urban | 26 | 51.65 | 22.35 |  |  |  |
| Rural | 20.18 | 68.38 | 11.44 |  |  |  |

LDHS: Lesotho Demographic and Health Survey, *Derived from chi-square test.
