# Supplementary material for: Factors associated with and socioeconomic inequalities in underweight, overweight and obesity among adults aged 18–49 years in Lesotho: Evidence from the 2023–2024 Demographic and Health Survey
Source: PLOS Glob Public Health. 2026 Jan 20;6(1):e0005555. doi: 10.1371/journal.pgph.0005555 (PMC12818733; doi:10.1371/journal.pgph.0005555)
Supplement: S2 Table — (DOCX) [file pgph.0005555.s002.docx]

**S2 Table: Prevalence of underweight, normal weight, and overweight/obesity by socio-demographic characteristics among female participants aged 18–49 years, LDHS 2023–2024**

| **Variables** | **BMI (%)** | | | **Pearson χ² (df)** | **Design-based F (df1, df2)** | ***P*-value*** |
| --- | --- | --- | --- | --- | --- | --- |
|  | **Underweight** | **Normal BMI** | **Overweight/Obesity** |  |  |  |
| **Age Group** |  |  |  | χ²(4)=546.10 | F(3.84,1423.89)=40.74 | <0.001 |
| 18–29 | 10.26 | 51.87 | 37.87 |  |  |  |
| 30–39 | 7.39 | 23.45 | 69.16 |  |  |  |
| 40–49 | 7.17 | 24.09 | 68.74 |  |  |  |
| **Education** |  |  |  | χ²(4)=25.22 | F(3.06,1135.31)=1.13 | >0.05 |
| No education or primary | 7.84 | 34.71 | 57.45 |  |  |  |
| Secondary | 8.46 | 39.16 | 52.38 |  |  |  |
| Higher | 10.35 | 31.61 | 58.04 |  |  |  |
| **Marital Status** |  |  |  | χ²(4)=451.96 | F(3.83,1422.12)=38.74 | <0.001 |
| Never married | 12.56 | 54.11 | 33.33 |  |  |  |
| Married | 6.39 | 28.57 | 65.04 |  |  |  |
| Widowed/Divorce/Separated | 8.9 | 30 | 61.11 |  |  |  |
| **Wealth Index** |  |  |  | χ²(8)=167.45 | F(5.95,2206.44)=5.19 | <0.001 |
| Poorest | 7.32 | 49.8 | 42.89 |  |  |  |
| Poorer | 5.92 | 43.19 | 50.9 |  |  |  |
| Middle | 9.9 | 39.27 | 50.83 |  |  |  |
| Richer | 7.98 | 34.83 | 57.19 |  |  |  |
| Richest | 10.72 | 25.16 | 64.13 |  |  |  |
| **Ecological Zone** |  |  |  | χ²(6)=51.74 | F(5.13,1904.32)=4.45 | <0.01 |
| Lowlands | 10 | 35.07 | 54.93 |  |  |  |
| Foothills | 3.62 | 47.27 | 49.11 |  |  |  |
| Mountains | 5.09 | 37.67 | 57.24 |  |  |  |
| Senqu River Valley | 6.31 | 40 | 53.69 |  |  |  |
| **Region of Residence** |  |  |  | χ²(18)=116.29 | F(11.71,4345.28)=3.45 | <0.01 |
| Butha-Buthe | 4.27 | 33.94 | 61.79 |  |  |  |
| Leribe | 7.51 | 37.34 | 55.14 |  |  |  |
| Berea | 5 | 36.96 | 58.04 |  |  |  |
| Maseru | 13.77 | 33.84 | 52.39 |  |  |  |
| Mafeteng | 7.4 | 43.65 | 48.95 |  |  |  |
| Mohale's Hoek | 5.6 | 36.79 | 57.61 |  |  |  |
| Quthing | 5.83 | 35.88 | 58.29 |  |  |  |
| Qacha's Nek | 5.39 | 35.43 | 59.17 |  |  |  |
| Mokhotlong | 4.54 | 39.75 | 55.72 |  |  |  |
| Thaba-Tseka | 6.24 | 43.57 | 50.19 |  |  |  |
| **Place of Residence** |  |  |  | χ²(2)=68.19 | F(1.96,727.60)=8.74 | <0.01 |
| Urban | 10.14 | 30.89 | 58.97 |  |  |  |
| Rural | 7.37 | 41.44 | 51.19 |  |  |  |

LDHS: Lesotho Demographic and Health Survey, *Derived from chi-square test.
