# Supplementary material for: Factors associated with and socioeconomic inequalities in underweight, overweight and obesity among adults aged 18–49 years in Lesotho: Evidence from the 2023–2024 Demographic and Health Survey
Source: PLOS Glob Public Health. 2026 Jan 20;6(1):e0005555. doi: 10.1371/journal.pgph.0005555 (PMC12818733; doi:10.1371/journal.pgph.0005555)
Supplement: S3 Table — (DOCX) [file pgph.0005555.s003.docx]

**S3 Table: Crude and adjusted odds ratios for correlates of underweight and overweight/obesity among male participants aged 18–49 years, LDHS 2023–2024**

| **Variables** | **Underweight** | | | | **Overweight/Obesity** | | | |
| --- | --- | --- | --- | --- | --- | --- | --- | --- |
|  | **COR (95% CI)** | ***p*-value** | **AOR (95% CI)** | ***p*-value** | **COR (95% CI)** | ***p*-value** | **AOR (95% CI)** | ***p*-value** |
| **Age Group** |  |  |  |  |  |  |  |  |
| 18–29 | Ref |  | Ref |  | Ref |  | Ref |  |
| 30–39 | 1.33 (0.99-1.79) | >0.05 | 1.44 (0.98–2.10) | >0.05 | 3.33 (2.26-4.91) | <0.001 | **2.25 (1.48-3.41)** | <0.001 |
| 40–49 | 1.26 (0.91-1.75) | >0.05 | 1.45 (0.94–2.23) | >0.05 | 4.07 (2.71-6.12) | <0.001 | **3.15 (1.92-5.15)** | <0.001 |
| **Education** |  |  |  |  |  |  |  |  |
| No education or primary | Ref |  | Ref |  | Ref |  | Ref |  |
| Secondary | 1.05 (0.75-1.47) | >0.05 | 0.97 (0.67–1.39) | >0.05 | 1.46 (1.07-1.99) | <0.05 | 1.12 (0.74-1.70) | >0.05 |
| Higher | 1.52 (0.99-2.35) | >0.05 | 1.30 (0.78–2.20) | >0.05 | 4.04 (2.69-6.06) | <0.001 | **1.77 (1.02-3.06)** | >0.05 |
| **Marital Status** |  |  |  |  |  |  |  |  |
| Never married | Ref |  | Ref |  | Ref |  | Ref |  |
| Married | 0.93 (0.73-1.19) | >0.05 | 0.78 (0.57–1.07) | >0.05 | 3.62 (2.51-5.22) | <0.001 | **2.13 (1.44-3.16)** | <0.01 |
| Widowed/Divorce/Separated | 1.03 (0.71-1.50) | >0.05 | 0.78 (0.49–1.25) | >0.05 | 2.08 (1.26-3.44) | <0.01 | 1.33 (0.75-2.37) | >0.05 |
| **Wealth Index** |  |  |  |  |  |  |  |  |
| Poorest | Ref |  | Ref |  | Ref |  | Ref |  |
| Poorer | 1.48 (1.03-2.13) | <0.05 | 0.94 (0.66–1.35) | >0.05 | 1.00 (0.53-1.89) | >0.05 | 1.16 (0.63-2.14) | >0.05 |
| Middle | 1.33 (0.89-2.00) | >0.05 | 0.67 (0.43–1.05) | >0.05 | 2.12 (1.31-3.42) | <0.01 | **2.81 (1.63-4.84)** | <0.001 |
| Richer | 1.72 (1.11-2.67) | <0.05 | 0.70 (0.41–1.19) | >0.05 | 4.79 (3.09-7.42) | <0.001 | **5.49 (3.03-9.96)** | <0.001 |
| Richest | 1.61 (1.05-2.45) | <0.05 | 0.56 (0.30–1.05) | >0.05 | 7.82 (5.03-12.17) | <0.001 | **6.76 (3.28-13.92)** | <0.001 |
| **Ecological Zone** |  |  |  |  |  |  |  |  |
| Lowlands | Ref |  | Ref |  | Ref |  | Ref |  |
| Foothills | 0.88 (0.52-1.48) | >0.05 | 0.95 (0.56–1.62) | >0.05 | 0.30 (0.16-0.56) | <0.001 | 0.88 (0.43-1.82) | >0.05 |
| Mountains | 0.40 (0.29-0.54) | <0.001 | **0.48 (0.29–0.79)** | <0.01 | 0.53 (0.38-0.74) | <0.001 | 1.02 (0.45-2.32) | >0.05 |
| Senqu River Valley | 0.49 (0.33-0.72) | <0.001 | 0.58 (0.30–1.13) | >0.05 | 0.53 (0.34-0.81) | <0.01 | 0.88 (0.36-2.12) | >0.05 |
| **Region of Residence** |  |  |  |  |  |  |  |  |
| Butha-Buthe | Ref |  | Ref |  | Ref |  | Ref |  |
| Leribe | 1.20 (0.75-1.93) | >0.05 | 1.24 (0.76–2.03) | >0.05 | 0.87 (0.51-1.49) | >0.05 | 0.75 (0.46-1.22) | >0.05 |
| Berea | 1.23 (0.71-2.12) | >0.05 | 1.20 (0.68–2.12) | >0.05 | 1.81 (1.04-3.17) | <0.05 | 1.47 (0.83-2.62) | >0.05 |
| Maseru | 1.78 (1.11-2.86) | <0.05 | **1.78 (1.08–2.94)** | <0.05 | 1.59 (0.95-2.66) | >0.05 | 1.02 (0.63-1.65) | >0.05 |
| Mafeteng | 1.09 (0.70-1.71) | >0.05 | 1.10 (0.67–1.78) | >0.05 | 1.12 (0.65-1.94) | >0.05 | 1.15 (0.67-1.96) | >0.05 |
| Mohale's Hoek | 0.83 (0.48-1.42) | >0.05 | 0.93 (0.51–1.67) | >0.05 | 0.86 (0.44-1.66) | >0.05 | 1.20 (0.58-2.48) | >0.05 |
| Quthing | 0.86 (0.50-1.46) | >0.05 | 1.33 (0.62–2.87) | >0.05 | 0.93 (0.51-1.72) | >0.05 | 1.44 (0.54-3.81) | >0.05 |
| Qacha's Nek | 0.75 (0.38-1.49) | >0.05 | 1.38 (0.58–3.27) | >0.05 | 1.11 (0.61-2.02) | >0.05 | 1.04 (0.41-2.59) | >0.05 |
| Mokhotlong | 0.93 (0.53-1.63) | >0.05 | 1.76 (0.87–3.56) | >0.05 | 1.00 (0.54-1.84) | >0.05 | 1.47 (0.59-3.64) | >0.05 |
| Thaba-Tseka | 0.23 (0.11-0.46) | <0.001 | 0.43 (0.19–0.96) | <0.05 | 0.71 (0.38-1.31) | >0.05 | 1.50 (0.62-3.62) | >0.05 |
| **Place of Residence** |  |  |  |  |  |  |  |  |
| Urban | Ref |  | Ref |  | Ref |  | Ref |  |
| Rural | 0.59 (0.43-0.80) | <0.01 | 0.65 (0.41–1.04) | >0.05 | 0.39 (0.28-0.53) | <0.001 | 0.89 (0.63-1.26) | >0.05 |

*AOR: Adjusted Odds Ratio; COR: Crude Odds Ratio; CI: Confidence Interval; LDHS: Lesotho Demographic and Health Survey. Statistically significant AORs (p<0.05) are presented in bold.*
