# Supplementary material for: Factors associated with and socioeconomic inequalities in underweight, overweight and obesity among adults aged 18–49 years in Lesotho: Evidence from the 2023–2024 Demographic and Health Survey
Source: PLOS Glob Public Health. 2026 Jan 20;6(1):e0005555. doi: 10.1371/journal.pgph.0005555 (PMC12818733; doi:10.1371/journal.pgph.0005555)
Supplement: S4 Table — (DOCX) [file pgph.0005555.s004.docx]

**S4 Table: Crude and adjusted odds ratios for correlates of underweight and overweight/obesity among female participants aged 18–49 years, LDHS 2023–2024**

|  | **Underweight** | | | | **Overweight/Obesity** | | | |
| --- | --- | --- | --- | --- | --- | --- | --- | --- |
| **Variables** | **COR (95% CI)** | ***p*-value** | **AOR (95% CI)** | ***p*-value** | **COR (95% CI)** | ***p*-value** | **AOR (95% CI)** | ***p*-value** |
| **Age Group** |  |  |  |  |  |  |  |  |
| 18–29 | Ref |  | Ref |  | Ref |  | Ref |  |
| 30–39 | 1.59 (1.04-2.43) | <0.05 | 1.58 (1.00-2.51) | <0.05 | 4.04 (3.18-5.13) | <0.001 | **2.96 (2.28-3.83)** | <0.001 |
| 40–49 | 1.50 (0.91-2.49) | >0.05 | 1.46 (0.88-2.42) | >0.05 | 3.91 (2.99-5.12) | <0.001 | **2.79 (2.03-3.84)** | <0.001 |
| **Education** |  |  |  |  |  |  |  |  |
| No education or primary | Ref |  | Ref |  | Ref |  | Ref |  |
| Secondary | 0.96 (0.59-1.54) | >0.05 | 0.83 (0.49-1.39) | >0.05 | 0.81 (0.65-1.00) | >0.05 | 1.00 (0.77-1.30) | >0.05 |
| Higher | 1.45 (0.59-3.54) | >0.05 | 0.76 (0.28-2.04) | >0.05 | 1.11 (0.73-1.69) | >0.05 | 0.89 (0.57-1.40) | >0.05 |
| **Marital Status** |  |  |  |  |  |  |  |  |
| Never married | Ref |  | Ref |  | Ref |  | Ref |  |
| Married | 0.96 (0.66-1.41) | >0.05 | 0.91 (0.57-1.46) | >0.05 | 3.70 (2.99-4.57) | <0.001 | **2.80 (2.22-3.52)** | <0.001 |
| Widowed/Divorce/Separated | 1.28 (0.76-2.16) | >0.05 | 1.20 (0.70-2.06) | >0.05 | 3.31 (2.46-4.44) | <0.001 | **2.46 (1.78-3.41)** | <0.001 |
| **Wealth Index** |  |  |  |  |  |  |  |  |
| Poorest | Ref |  | Ref |  | Ref |  | Ref |  |
| Poorer | 0.93 (0.51-1.72) | >0.05 | 0.76 (0.38-1.51) | >0.05 | 1.37 (1.05-1.79) | <0.05 | **1.94 (1.42-2.65)** | <0.001 |
| Middle | 1.72 (0.84-3.51) | >0.05 | 1.07 (0.52-2.22) | >0.05 | 1.50 (1.11-2.04) | <0.01 | **2.57 (1.84-3.61)** | <0.001 |
| Richer | 1.56 (0.91-2.68) | >0.05 | 0.89 (0.41-1.94) | >0.05 | 1.91 (1.40-2.59) | <0.001 | **3.15 (2.00-4.98)** | <0.001 |
| Richest | 2.90 (1.64-5.12) | <0.001 | 1.61 (0.74-3.53) | >0.05 | 2.96 (2.21-3.97) | <0.001 | **5.14 (3.31-8.00)** | <0.001 |
| **Ecological Zone** |  |  |  |  |  |  |  |  |
| Lowlands | Ref |  | Ref |  | Ref |  | Ref |  |
| Foothills | 0.27 (0.12-0.61) | <0.01 | 0.34 (0.14-0.87) | <0.05 | 0.66 (0.48-0.92) | <0.05 | 1.28 (0.88-1.86) | >0.05 |
| Mountains | 0.47 (0.30-0.75) | <0.01 | 0.77 (0.28-2.13) | >0.05 | 0.97 (0.75-1.25) | >0.05 | **3.10 (1.42-6.77)** | <0.01 |
| Senqu River Valley | 0.55 (0.32-0.96) | <0.05 | 0.81 (0.24-2.79) | >0.05 | 0.86 (0.64-1.14) | >0.05 | 1.78 (0.92-3.47) | >0.05 |
| **Region of Residence** |  |  |  |  |  |  |  |  |
| Butha-Buthe | Ref |  | Ref |  | Ref |  | Ref |  |
| Leribe | 1.60 (0.75-3.40) | >0.05 | 1.26 (0.59-2.71) | >0.05 | 0.81 (0.58-1.13) | >0.05 | **0.69 (0.48-0.99)** | >0.05 |
| Berea | 1.08 (0.47-2.49) | >0.05 | 0.82 (0.35-1.92) | >0.05 | 0.86 (0.65-1.15) | >0.05 | **0.70 (0.50-0.97)** | >0.05 |
| Maseru | 3.24 (1.51-6.93) | >0.05 | **2.38 (1.09-5.18)** | <0.05 | 0.85 (0.59-1.22) | <0.01 | **0.67 (0.45-0.99)** | >0.05 |
| Mafeteng | 1.35 (0.61-2.99) | <0.05 | 1.08 (0.46-2.56) | >0.05 | 0.62 (0.42-0.89) | >0.05 | **0.55 (0.37-0.82)** | <0.01 |
| Mohale's Hoek | 1.21 (0.46-3.21) | >0.05 | 1.07 (0.42-2.71) | >0.05 | 0.86 (0.62-1.19) | >0.05 | 0.81 (0.52-1.26) | >0.05 |
| Quthing | 1.29 (0.54-3.12) | >0.05 | 1.25 (0.30-5.32) | >0.05 | 0.89 (0.59-1.34) | >0.05 | 0.69 (0.34-1.42) | >0.05 |
| Qacha's Nek | 1.21 (0.49-3.00) | >0.05 | 1.21 (0.32-4.51) | >0.05 | 0.92 (0.63-1.33) | >0.05 | 0.53 (0.26-1.11) | >0.05 |
| Mokhotlong | 0.91 (0.35-2.34) | >0.05 | 0.91 (0.24-3.42) | >0.05 | 0.77 (0.50-1.19) | >0.05 | **0.38 (0.16-0.86)** | <0.05 |
| Thaba-Tseka | 1.14 (0.48-2.73) | <0.05 | 1.12 (0.32-3.96) | >0.05 | 0.63 (0.45-0.89) | >0.05 | **0.35 (0.16-0.74)** | <0.01 |
| **Place of Residence** |  |  |  |  |  |  |  |  |
| Urban | Ref |  | Ref |  | Ref |  | Ref |  |
| Rural | 0.54 (0.37-0.80) | <0.01 | 0.93 (0.54-1.58) | >0.05 | 0.65 (0.52-0.81) | <0.001 | 0.85 (0.55-1.30) | >0.05 |

*AOR: Adjusted Odds Ratio; COR: Crude Odds Ratio; CI: Confidence Interval; LDHS: Lesotho Demographic and Health Survey. Statistically significant AORs (p<0.05) are presented in bold.*
