# Supplementary material for: Factors associated with and socioeconomic inequalities in underweight, overweight and obesity among adults aged 18–49 years in Lesotho: Evidence from the 2023–2024 Demographic and Health Survey
Source: PLOS Glob Public Health. 2026 Jan 20;6(1):e0005555. doi: 10.1371/journal.pgph.0005555 (PMC12818733; doi:10.1371/journal.pgph.0005555)
Supplement: S5 Table — (DOCX) [file pgph.0005555.s005.docx]

**S5 Table: Socioeconomic inequalities in underweight among male participants, LDHS 2023–2024**

| **Variable** | **Q1 (%)** | **Q5 (%)** | **Q5-Q1 (%)** | **Q5/Q1** | **Index Value** | **Standard Error** | ***P*-value** |
| --- | --- | --- | --- | --- | --- | --- | --- |
| **Total** | 19.35 | 20.16 | 0.81 | 1.04 | 0.003 | 0.0191 | >0.05 |
| **Age Group** |  |  |  |  |  |  |  |
| 18–29 | 17.66 | 18.99 | 1.33 | 1.08 | 0.0116 | 0.0286 | >0.05 |
| 30–39 | 23.64 | 20.42 | -3.22 | 0.86 | 0.004 | 0.0349 | >0.05 |
| 40–49 | 17.32 | 21.48 | 4.16 | 1.24 | -0.0075 | 0.0378 | >0.05 |
| **Sex** |  |  |  |  |  |  |  |
| Male | 19.40 | 23.62 | 4.22 | 1.22 | 0.0324 | 0.0273 | >0.05 |
| Female | 19.03 | 15.59 | -3.44 | 0.82 | -0.0437 | 0.0306 | >0.05 |
| **Education** | 20.39 | 24.14 | 3.75 | 1.18 | 0.0813 | 0.0544 | >0.05 |
| No education or primary |  |  |  |  |  |  |  |
| Secondary | 20.22 | 19.79 | -0.43 | 0.98 | -0.0166 | 0.0295 | >0.05 |
| Higher | 20.40 | 18.14 | -2.26 | 0.89 | -0.0008 | 0.0271 | >0.05 |
| **Marital Status** | 11.54 | 40.84 | 29.30 | 3.54 | 0.1161 | 0.0601 | >0.05 |
| Never married |  |  |  |  |  |  |  |
| Married | 24.65 | 20.78 | -3.87 | 0.84 | -0.0425 | 0.0273 | >0.05 |
| Widowed/Divorce/Separated | 36.94 | 42.94 | 6.00 | 1.16 | -0.2381 | 0.0694 | <0.001 |
| **Ecological Zone** | 13.20 | 5.17 | -8.03 | 0.39 | -0.0349 | 0.0293 | >0.05 |
| Lowlands | 13.15 | 9.61 | -3.54 | 0.73 | 0.0387 | 0.0464 | >0.05 |
| Foothills |  |  |  |  |  |  |  |
| Mountains | 17.83 | 18.54 | 0.71 | 1.04 | 0.0141 | 0.0576 | >0.05 |
| Senqu River Valley | 22.59 | 18.37 | -4.22 | 0.81 | -0.0742 | 0.054 | >0.05 |
| **Region of Residence** | 22.02 | 14.59 | -7.43 | 0.66 | -0.1151 | 0.0545 | <0.05 |
| Butha-Buthe | 45.25 | 25.60 | -19.65 | 0.57 | -0.017 | 0.0565 | >0.05 |
| Leribe | 21.84 | 21.37 | -0.47 | 0.98 | -0.0059 | 0.0598 | >0.05 |
| Berea | 22.60 | 0.00 | -22.6 | 0 | -0.1446 | 0.0616 | <0.05 |
| Maseru | 15.08 | 19.20 | 4.12 | 1.27 | 0.0511 | 0.0611 | >0.05 |
| Mafeteng | 14.22 | 10.21 | -4.01 | 0.72 | -0.0751 | 0.0624 | >0.05 |
| Mohale's Hoek | 17.42 | 0.00 | -17.42 | 0 | -0.0091 | 0.0594 | >0.05 |
| Quthing | 6.49 | 0.00 | -6.49 | 0 | 0.0013 | 0.0331 | >0.05 |
| Qacha's Nek |  |  |  |  |  |  |  |
| Mokhotlong | 19.50 | 21.00 | 1.5 | 1.08 | −0.085 | 0.034 | <0.05 |
| Thaba-Tseka | 19.30 | 17.40 | −1.5 | 0.9 | −0.011 | 0.023 | >0.05 |
| **Place of Residence** |  |  |  |  |  |  |  |
| Urban | 10.21 | 14.84 | 4.63 | 1.45 | -0.054 | 0.020 | <0.01 |
| Rural | 13.54 | 13.50 | -0.04 | 1.00 | -0.003 | 0.013 | >0.05 |

*LDHS: Lesotho Demographic and Health Survey*
