# Supplementary material for: Factors associated with and socioeconomic inequalities in underweight, overweight and obesity among adults aged 18–49 years in Lesotho: Evidence from the 2023–2024 Demographic and Health Survey
Source: PLOS Glob Public Health. 2026 Jan 20;6(1):e0005555. doi: 10.1371/journal.pgph.0005555 (PMC12818733; doi:10.1371/journal.pgph.0005555)
Supplement: S6 Table — (DOCX) [file pgph.0005555.s006.docx]

**S6 Table: Socioeconomic inequalities in overweight/obesity among male participants, LDHS 2023–2024**

| **Variable** | **Q1 (%)** | **Q5 (%)** | **Q5-Q1 (%)** | **Q5/Q1** | **Index Value** | **Standard Error** | **P-value** |
| --- | --- | --- | --- | --- | --- | --- | --- |
| **Total** | 6.23 | 31.57 | 25.34 | 5.07 | 0.2116 | 0.0162 | <0.001 |
| **Age Group** |  |  |  |  |  |  |  |
| 18–29 | 3.98 | 14.14 | 10.16 | 3.55 | 0.0843 | 0.0184 | <0.001 |
| 30–39 | 6.42 | 44.49 | 38.07 | 6.93 | 0.292 | 0.0318 | <0.001 |
| 40–49 | 9.16 | 42.00 | 32.84 | 4.58 | 0.3296 | 0.0374 | <0.001 |
| **Sex** |  |  |  |  |  |  |  |
| Male | 6.97 | 25.35 | 18.38 | 3.63 | 0.121 | 0.0203 | <0.001 |
| Female | 1.91 | 19.45 | 17.54 | 10.20 | 0.1516 | 0.0261 | <0.001 |
| **Education** | 0.00 | 45.64 | 45.64 | – | 0.2866 | 0.0573 | <0.001 |
| No education or primary |  |  |  |  |  |  |  |
| Secondary | 4.52 | 13.46 | 8.94 | 2.98 | 0.0654 | 0.0185 | <0.001 |
| Higher | 8.35 | 47.71 | 39.36 | 5.72 | 0.3417 | 0.0276 | <0.001 |
| **Marital Status** | 3.08 | 26.94 | 23.86 | 8.74 | 0.2501 | 0.049 | <0.001 |
| Never married |  |  |  |  |  |  |  |
| Married | 3.70 | 31.28 | 27.58 | 8.45 | 0.216 | 0.0236 | <0.001 |
| Widowed/Divorce/Separated | 2.53 | 0.00 | -2.53 | 0.00 | 0.0787 | 0.0394 | >0.05 |
| **Ecological Zone** | 9.22 | 41.56 | 32.34 | 4.51 | 0.1183 | 0.0287 | <0.001 |
| Lowlands | 3.35 | 39.02 | 35.67 | 11.64 | 0.2245 | 0.04 | <0.001 |
| Foothills |  |  |  |  |  |  |  |
| Mountains | 2.28 | 37.71 | 35.43 | 16.47 | 0.2236 | 0.048 | <0.001 |
| Senqu River Valley | 6.22 | 21.75 | 15.53 | 0.28 | 0.1487 | 0.0406 | <0.001 |
| **Region of Residence** | 2.86 | 35.88 | 33.02 | 11.78 | 0.2586 | 0.0535 | <0.001 |
| Butha-Buthe | 0.00 | 31.23 | 31.23 | — | 0.2317 | 0.0462 | <0.001 |
| Leribe | 4.28 | 27.98 | 23.70 | 6.53 | 0.1817 | 0.0513 | <0.001 |
| Berea | 0.00 | 48.92 | 48.92 | — | 0.2938 | 0.0506 | <0.001 |
| Maseru | 6.53 | 35.19 | 28.66 | 5.39 | 0.1582 | 0.0537 | <0.01 |
| Mafeteng | 11.19 | 15.33 | 4.14 | 1.37 | 0.0979 | 0.0632 | >0.05 |
| Mohale's Hoek | 9.19 | 59.95 | 50.76 | 6.53 | 0.1898 | 0.0514 | <0.001 |
| Quthing | 11.33 | 35.29 | 23.96 | 3.12 | 0.0399 | 0.0468 | >0.05 |
| Qacha's Nek |  |  |  |  |  |  |  |
| Mokhotlong | 5.80 | 32.20 | 26.50 | 5.59 | 0.204 | 0.032 | <0.001 |
| Thaba-Tseka | 6.20 | 29.40 | 23.10 | 4.71 | 0.138 | 0.018 | <0.001 |
| **Place of Residence** |  |  |  |  |  |  |  |
| Urban | 6.23 | 31.57 | 25.34 | 5.07 | 0.2116 | 0.0162 | <0.001 |
| Rural | 3.98 | 14.14 | 10.16 | 3.55 | 0.0843 | 0.0184 | <0.001 |

*LDHS: Lesotho Demographic and Health Survey*
