# Supplementary material for: Factors associated with and socioeconomic inequalities in underweight, overweight and obesity among adults aged 18–49 years in Lesotho: Evidence from the 2023–2024 Demographic and Health Survey
Source: PLOS Glob Public Health. 2026 Jan 20;6(1):e0005555. doi: 10.1371/journal.pgph.0005555 (PMC12818733; doi:10.1371/journal.pgph.0005555)
Supplement: S7 Table — (DOCX) [file pgph.0005555.s007.docx]

**S7 Table: Socioeconomic inequalities in underweight among female participants, LDHS 2023–2024**

| **Variable** | **Q1 (%)** | **Q5 (%)** | **Q5-Q1 (%)** | **Q5/Q1** | **Index Value** | **Standard Error** | ***P*-value** |
| --- | --- | --- | --- | --- | --- | --- | --- |
| **Total** | 7.32 | 10.72 | 3.40 | 1.47 | 0.027 | 0.0121 | <0.05 |
| **Age Group** |  |  |  |  |  |  |  |
| 18–29 | 10.46 | 12.45 | 1.99 | 1.19 | 0.0306 | 0.019 | >0.05 |
| 30–39 | 3.00 | 11.57 | 8.57 | 3.86 | 0.0419 | 0.0205 | <0.05 |
| 40–49 | 5.23 | 7.13 | 1.90 | 1.36 | 0.0088 | 0.0231 | >0.05 |
| **Sex** |  |  |  |  |  |  |  |
| Male | 6.77 | 7.98 | 1.21 | 1.18 | 0.0283 | 0.0204 | >0.05 |
| Female | 8.29 | 9.40 | 1.11 | 1.13 | 0.0085 | 0.0163 | >0.05 |
| **Education** | 0.00 | 12.79 | 12.79 | – | 0.0742 | 0.0349 | <0.05 |
| No education or primary |  |  |  |  |  |  |  |
| Secondary | 10.77 | 16.53 | 5.76 | 1.54 | 0.04 | 0.0262 | >0.05 |
| Higher | 7.64 | 7.46 | -0.18 | 0.98 | 0.0105 | 0.0141 | >0.05 |
| **Marital Status** | 2.11 | 9.39 | 7.28 | 4.45 | 0.0536 | 0.0312 | >0.05 |
| Never married |  |  |  |  |  |  |  |
| Married | 9.25 | 11.25 | 2.00 | 1.22 | 0.0107 | 0.0177 | >0.05 |
| Widowed/Divorce/Separated | 3.44 | 0.00 | -3.44 | 0.00 | 0.0201 | 0.0294 | >0.05 |
| **Ecological Zone** | 7.66 | 0.00 | -7.66 | 0.00 | -0.0536 | 0.0182 | <0.01 |
| Lowlands | 8.27 | 4.88 | -3.39 | 0.59 | -0.0336 | 0.0292 | >0.05 |
| Foothills |  |  |  |  |  |  |  |
| Mountains | 1.47 | 6.03 | 4.56 | 4.11 | 0.0231 | 0.0263 | >0.05 |
| Senqu River Valley | 7.42 | 4.41 | -3.01 | 0.59 | -0.0306 | 0.032 | >0.05 |
| **Region of Residence** | 5.62 | 8.79 | 3.17 | 1.56 | 0.0519 | 0.027 | >0.05 |
| Butha-Buthe | 7.75 | 15.81 | 8.06 | 2.04 | 0.0247 | 0.0396 | >0.05 |
| Leribe | 18.05 | 8.44 | -9.61 | 0.47 | -0.0142 | 0.0385 | >0.05 |
| Berea | 8.07 | 0.00 | -8.07 | 0.00 | -0.0585 | 0.0358 | >0.05 |
| Maseru | 9.24 | 7.41 | -1.83 | 0.80 | -0.0258 | 0.0351 | >0.05 |
| Mafeteng | 10.01 | 0.00 | -10.01 | 0.00 | -0.0407 | 0.0352 | >0.05 |
| Mohale's Hoek | 7.33 | 0.00 | -7.33 | 0.00 | -0.0592 | 0.03 | <0.05 |
| Quthing | 6.96 | 0.00 | -6.96 | 0.00 | -0.0482 | 0.0332 | >0.05 |
| Qacha's Nek |  |  |  |  |  |  |  |
| Mokhotlong | 2.60 | 10.70 | 8.40 | 4.08 | −0.002 | 0.021 | >0.05 |
| Thaba-Tseka | 7.50 | 10.80 | 3.40 | 1.45 | 0.022 | 0.014 | >0.05 |
| **Place of Residence** |  |  |  |  |  |  |  |
| Urban | 10.21 | 14.84 | 4.63 | 1.45 | -0.054 | 0.020 | <0.01 |
| Rural | 13.54 | 13.50 | -0.04 | 1.00 | -0.003 | 0.013 | >0.05 |

*LDHS: Lesotho Demographic and Health Survey*
