# Supplementary material for: Factors associated with and socioeconomic inequalities in underweight, overweight and obesity among adults aged 18–49 years in Lesotho: Evidence from the 2023–2024 Demographic and Health Survey
Source: PLOS Glob Public Health. 2026 Jan 20;6(1):e0005555. doi: 10.1371/journal.pgph.0005555 (PMC12818733; doi:10.1371/journal.pgph.0005555)
Supplement: S8 Table — (DOCX) [file pgph.0005555.s008.docx]

**S8 Table: Socioeconomic inequalities in overweight/obesity among female participants in Lesotho, LDHS 2023-24**

| **Variable** | **Q1 (%)** | **Q5 (%)** | **Q5-Q1 (%)** | **Q5/Q1** | **Index Value** | **Standard Error** | **P-value** |
| --- | --- | --- | --- | --- | --- | --- | --- |
| **Total** | 42.89 | 64.13 | 21.24 | 1.50 | 0.1573 | 0.0212 | <0.001 |
| **Age Group** |  |  |  |  |  |  |  |
| 18–29 | 30.55 | 43.02 | 12.47 | 1.41 | 0.074 | 0.0304 | <0.05 |
| 30–39 | 51.29 | 80.16 | 28.87 | 1.56 | 0.2302 | 0.0353 | <0.001 |
| 40–49 | 58.94 | 74.77 | 15.83 | 1.27 | 0.1073 | 0.0413 | <0.01 |
| **Sex** |  |  |  |  |  |  |  |
| Male | 46.34 | 60.79 | 14.45 | 1.31 | 0.16 | 0.0371 | <0.001 |
| Female | 37.09 | 64.77 | 27.68 | 1.75 | 0.2052 | 0.0288 | <0.001 |
| **Education** | 66.40 | 64.19 | -2.21 | 0.97 | 0.1256 | 0.0565 | <0.05 |
| No education or primary |  |  |  |  |  |  |  |
| Secondary | 21.74 | 39.36 | 17.62 | 1.81 | 0.1243 | 0.0371 | <0.001 |
| Higher | 48.39 | 77.20 | 28.81 | 1.60 | 0.2044 | 0.0271 | <0.001 |
| **Marital Status** | 45.80 | 74.16 | 28.36 | 1.62 | 0.2029 | 0.0527 | <0.001 |
| Never married |  |  |  |  |  |  |  |
| Married | 24.79 | 63.32 | 38.53 | 2.55 | 0.1795 | 0.029 | <0.001 |
| Widowed/Divorce/Separated | 45.81 | 45.35 | -0.46 | 0.99 | 0.0051 | 0.0789 | >0.05 |
| **Ecological Zone** | 47.10 | 80.94 | 33.84 | 1.72 | 0.2004 | 0.0406 | <0.001 |
| Lowlands | 44.34 | 80.56 | 36.22 | 1.82 | 0.1883 | 0.0592 | <0.01 |
| Foothills |  |  |  |  |  |  |  |
| Mountains | 46.72 | 77.17 | 30.45 | 1.65 | 0.2283 | 0.0621 | <0.001 |
| Senqu River Valley | 25.99 | 66.48 | 40.49 | 2.56 | 0.2399 | 0.0592 | <0.001 |
| **Region of Residence** | 43.70 | 62.56 | 18.86 | 1.43 | 0.1387 | 0.0611 | <0.05 |
| Butha-Buthe | 48.74 | 60.80 | 12.06 | 1.25 | 0.1321 | 0.057 | <0.05 |
| Leribe | 12.44 | 62.05 | 49.61 | 4.99 | 0.2789 | 0.0714 | <0.001 |
| Berea | 46.37 | 69.25 | 22.88 | 1.49 | 0.1548 | 0.0766 | <0.05 |
| Maseru | 54.87 | 81.19 | 26.32 | 1.48 | 0.1113 | 0.0736 | >0.05 |
| Mafeteng | 41.59 | 85.38 | 43.79 | 2.05 | 0.2361 | 0.0752 | <0.01 |
| Mohale's Hoek | 47.27 | 94.38 | 47.11 | 2.00 | 0.1466 | 0.0715 | <0.05 |
| Quthing | 42.96 | 68.61 | 25.65 | 1.60 | 0.1786 | 0.0681 | <0.01 |
| Qacha's Nek |  |  |  |  |  |  |  |
| Mokhotlong | 32.70 | 67.00 | 34.30 | 2.05 | 0.191 | 0.034 | <0.001 |
| Thaba-Tseka | 43.20 | 54.20 | 11.00 | 1.25 | 0.085 | 0.027 | <0.01 |
| **Place of Residence** |  |  |  |  |  |  |  |
| Urban | 20.59 | 53.00 | 32.41 | 2.57 | 0.232 | 0.025 | <0.001 |
| Rural | 24.32 | 44.11 | 19.79 | 1.81 | 0.137 | 0.018 | <0.001 |

*LDHS: Lesotho Demographic and Health Survey*
