# Supplementary figures and images for: Factors associated with and socioeconomic inequalities in underweight, overweight and obesity among adults aged 18–49 years in Lesotho: Evidence from the 2023–2024 Demographic and Health Survey
Source: PLOS Glob Public Health. 2026 Jan 20;6(1):e0005555. doi: 10.1371/journal.pgph.0005555 (PMC12818733; doi:10.1371/journal.pgph.0005555)

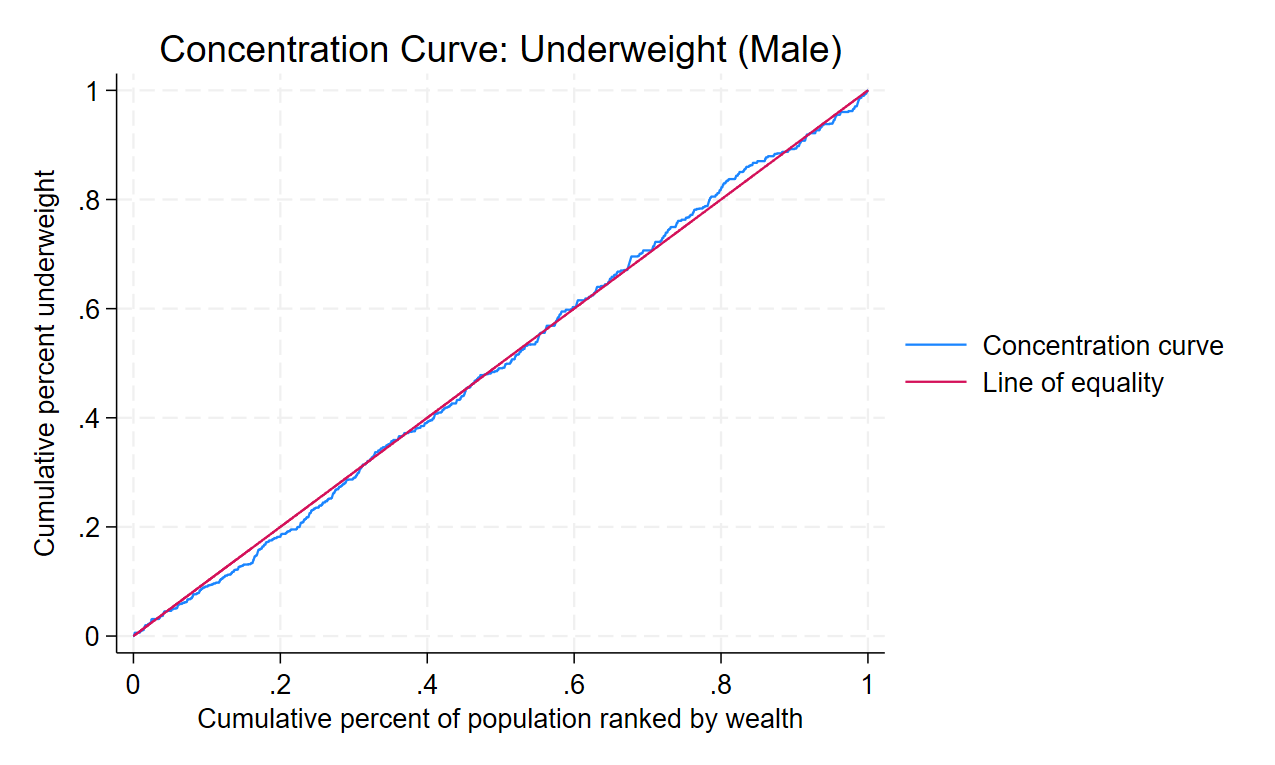

Supplement: S1 Fig — (TIF) [file pgph.0005555.s009.tif]

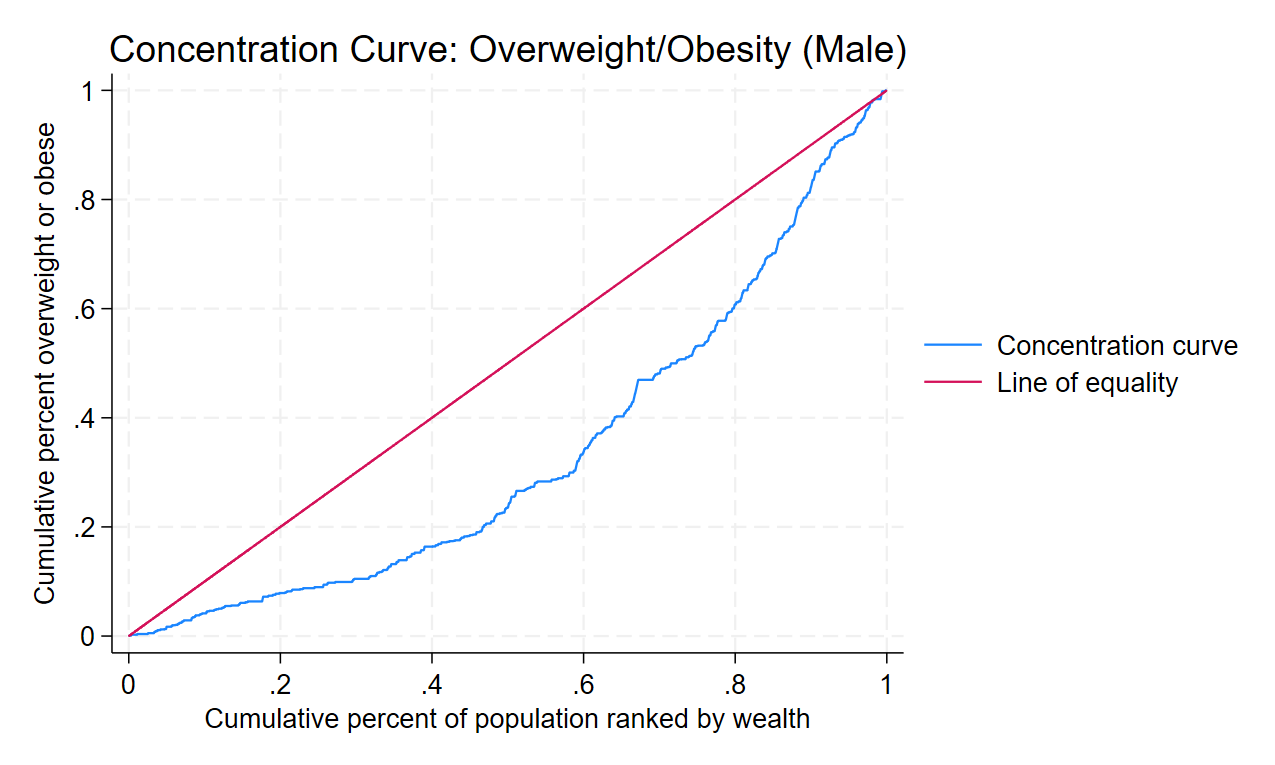

Supplement: S2 Fig — (TIF) [file pgph.0005555.s010.tif]

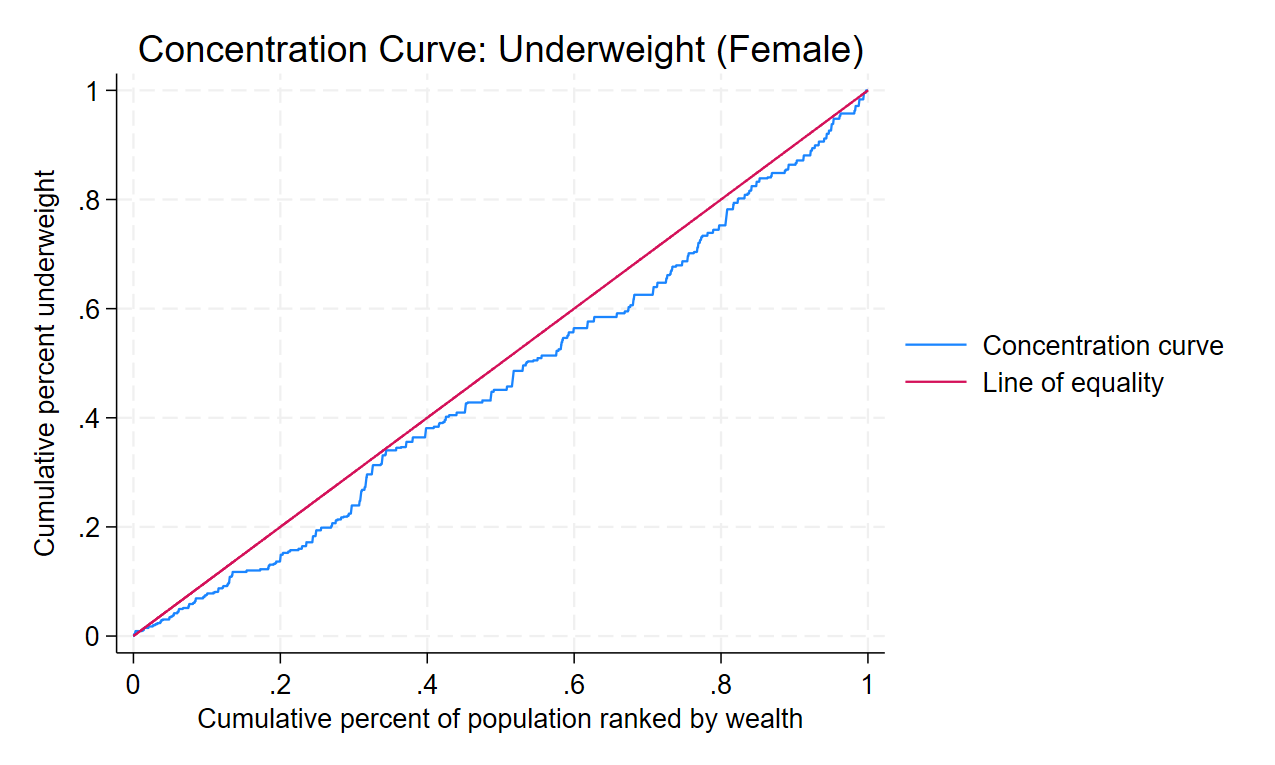

Supplement: S3 Fig — (TIF) [file pgph.0005555.s011.tif]

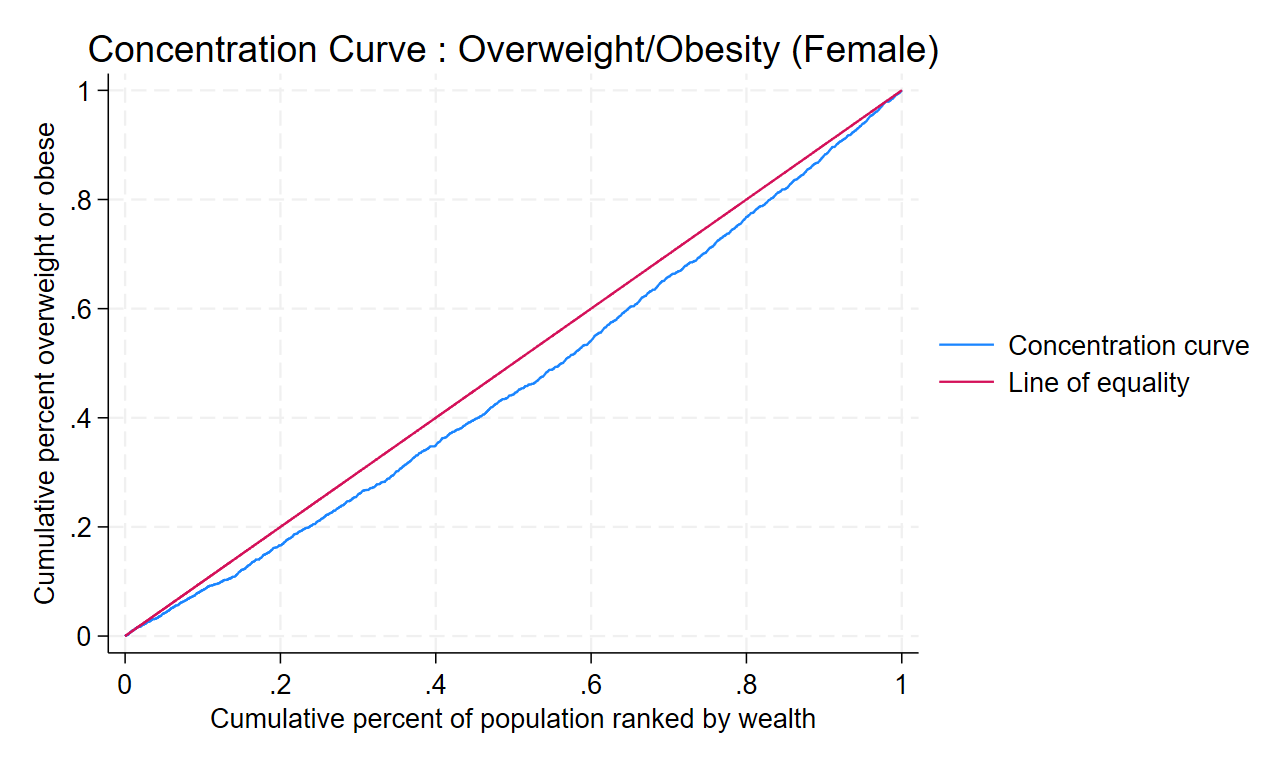

Supplement: S4 Fig — (TIF) [file pgph.0005555.s012.tif]
