# Supplementary material for: Factors associated with and socioeconomic inequalities in underweight, overweight and obesity among adults aged 18–49 years in Lesotho: Evidence from the 2023–2024 Demographic and Health Survey
Source: PLOS Glob Public Health. 2026 Jan 20;6(1):e0005555. doi: 10.1371/journal.pgph.0005555 (PMC12818733; doi:10.1371/journal.pgph.0005555)
Supplement: Attachment — Submitted filename: Response to Reviewers.docx [file pgph.0005555.s013.docx]

**Response to reviewers**

**Submitted Title:** Factors Associated with and Socioeconomic Inequalities in Underweight, Overweight and Obesity among Adults Aged 18–49 Years in Lesotho: Evidence from the 2023–2024 Demographic and Health Survey

**Reference Number:** PGPH-D-25-02639

*Journal Requirements:*

*1. Please ensure that your Ethics Statement is available in its entirety at the beginning of your*

*Methods section, under a subheading 'Ethics Statement'.*

**Response:** Thanks! We have placed the ethics statement in its entirety at the beginning of the

Methods section, under a subheading 'Ethics Statement'.

2. For studies involving third-party data, we encourage authors to share any data specific to their analyses that they can legally distribute. PLOS recognizes, however, that authors may be using third-party data they do not have the rights to share. When third-party data cannot be publicly shared, authors must provide all information necessary for interested researchers to apply to gain access to the data. (<https://journals.plos.org/plosone/s/data-availability#loc-acceptable-data-access-restrictions>) 
 
*For any third-party data that the authors cannot legally distribute, they should include the following information in their Data Availability Statement upon submission:*

*1) A description of the data set and the third-party source
 2) If applicable, verification of permission to use the data set
 3) Confirmation of whether the authors received any special privileges in accessing the data that other researchers would not have
 4) All necessary contact information others would need to apply to gain access to the data*

**Response:** Thanks! We have edited the data availability statement “This study used secondary data from the Demographic and Health Surveys Program. The Lesotho Demographic and Health Survey 2023–2024 dataset is collected and maintained by the Demographic and Health Surveys Program at ICF. The authors obtained permission to use the dataset by submitting a data request through the DHS Program website and received approval prior to conducting the analysis. The authors did not receive any special access privileges, and the data are available to all qualified researchers following the same application procedure. Interested investigators may request access through the DHS Program at <https://dhsprogram.com> by creating an account, submitting a brief research description, and specifying the required datasets. Further information can be obtained by contacting the DHS Program at [info@dhsprogram.com](mailto:info@dhsprogram.com) .”

*If the reviewer comments include a recommendation to cite specific previously published works, please review and evaluate these publications to determine whether they are relevant and should be cited. There is no requirement to cite these works unless the editor has indicated otherwise.* 

*Please review your reference list to ensure that it is complete and correct. If you have cited papers that have been retracted, please include the rationale for doing so in the manuscript text, or remove these references and replace them with relevant current references. Any changes to the reference list should be mentioned in the rebuttal letter that accompanies your revised manuscript. If you need to cite a retracted article, indicate the article’s retracted status in the References list and also include a citation and full reference for the retraction notice.*

**Response:** We have reviewed the reference list.

**Reviewer 1:**

*Thank you for submitting your manuscript titled “Factors Associated with and Socioeconomic Inequalities in Underweight, Overweight and Obesity among Adults Aged 18–49 Years in Lesotho: Evidence from the 2023–2024 Demographic and Health Survey.” The topic is timely and relevant, and I appreciate the effort invested in obtaining the data from DHS and presenting the findings. The paper addresses an important question on the growing trend of nutrition transition & epidemiological transition in sub-Saharan Africa, using recent data that includes various ages, genders and socioeconomic status, and has potential to contribute meaningfully to the discourse in this field.*

*Introduction: provides a good overview of the nutrition transition and Lesotho's unique circumstances
Methods: includes sufficient description of participant selection criteria & sampling methods (with original DHS resource linked) and data analysis, including rationale for methods chosen.
Results: good reporting of outcomes of analyses without overstating any effects. The writing here (as well as elsewhere in the paper, could benefit from reporting the OR and AOR in a consistent and clearer manner, e.g., (AOR=xx; 95%CI: xx-xx, p=xxx/p<0.05). Also, please review how the Chi-square tests are reported in your writing so that it is clearer to understand and differentiate from results of other statistical tests, and consider re-writing as, for example, χ(1) = 0.487, p = 0.485*

**Response:** Thank you for your comment. We appreciate this helpful suggestion. We have revised the Results section to ensure that all odds ratios and adjusted odds ratios follow a uniform reporting format. Throughout the manuscript, ORs and AORs are now presented as: AOR = xx; 95% CI: xx–xx; p < 0.05, with consistent punctuation and spacing.

We have also revised both the text and Table 1 to ensure clear, standardized reporting of chi-square tests. For each categorical comparison, the table now presents:

1. the uncorrected Pearson χ² statistic, with degrees of freedom in parentheses (e.g., χ²(4) = 338.74), and

2. the design-based F statistic, including numerator and denominator degrees of freedom (e.g., F(3.73, 1383.03) = 44.60), followed by the corresponding p-value.

We included in the manuscript “Both the uncorrected Pearson χ² statistics and the design-based F statistics indicated statistically significant associations between BMI status and all measured sociodemographic variables (p < 0.001; Table 1).”

*Discussion: well written with reference made to published work from other LMICs. Consider adding something on perimenopause and weight gain among older women, which could contribute to weight gain even with unchanged dietary behaviour, although this is very much under-researched in SSA. Adding these perspectives will make the paper more impactful.*

**Response:** Thanks! We have added the issues of perimenopause and weight gain among older women in the manuscript. We have discussed those in the subsequent section.

*Editing recommendations by line (please note that for OR/AOR and Chi-square results reporting, the authors are encouraged to review the entire document to check that the reporting styles are consistent throughout):*
*Line 19: change to* *'ever being married'*

**Response:** Thanks! We have edited as per suggestion.

Line 22: consider re-writing as (OR=xx; 95%CI: x-x, p<0.05), so that the reader can see the upper and lower bounds, and do same for line 23 (as well as other instances where OR/AOR are reported)

**Response:** Thanks! We have edited as per suggestion.

*Line 86: ages –* *aged*

**Response:** Thanks! We have edited as per suggestion.

*Line 87: ages – aged*

**Response:** Thanks! We have edited as per suggestion.

*Line 97: ages – aged*

**Response:** Thanks! We have edited as per suggestion.

*Line 100: ages – aged*

**Response:** Thanks! We have edited as per suggestion.

*Line 181, 182 & 183: include the p-values and OR when reporting the CI as well...consider revising as (**OR=1.49; 95%CI: 1.12-1.97, p...)*

**Response:** Thanks! We have edited as per suggestion.

*Line 185, 186, 187): consider re-writing to include p-values as well, so that it appears as (AOR=0.66; 95%CI: 0.51-0.87, p....)*

**Response:** Thanks! We have edited as per suggestion.

*Line 190, 191, 192 & 193): consider re-writing to include p-values as well, so that it appears as (AOR=0.66; 95%CI: 0.51-0.87, p....)*

**Response:** Thanks! We have edited as per suggestion.

*Line 232: recommend to highlight something regarding the quality of the food available. Whilst food availability has increased in many instances (which is varied rural vs urban), there have been a number of studies recently (2020-2025) that have used different datasets including World Bank LSMS, which indicate that the proportion of processed & ultra-processed foods, SSBs & foods away from home, has increased across SSA...this could be part of what is contributing to overweight/obesity?*

**Response:** Thank you for this important suggestion. We have included the following section “At the same time, recent evidence shows a marked shift in the quality of foods consumed across the region, with increasing intake of processed and ultra processed products, sugar sweetened beverages, and foods prepared away from home [25]. This transition in food systems, shaped by urbanization, rising incomes, and greater participation in the workforce, has intensified the nutrition transition and is increasingly linked to the growing double burden of malnutrition.”

*Line 248: Sub Saharan -* *sub-Saharan*

**Response:** Thanks! We have edited as per suggestion.

*Line 259: some clarification needed here - I'm not sure that 'nutrient-dense foods' would necessarily lead to weight gain [in any case, their consumption is encouraged], considering nutrient-dense foods are largely fruit, veg, pulses, nuts & seeds, and lean meats - unless you mean 'nutrient-dense, energy-dense'? Perhaps marriage would mean more HH income & therefore, more food security -> fewer meals missed ->more weight gained over time. Also, considering these are older women some of who may be experiencing perimenopause, characterised by weight gain even if dietary behaviours do not change much, might that be an additional factor at play here?*

**Response:** Thank you for this thoughtful comment. We agree that the phrase “nutrient-dense foods” was imprecise and could be misleading, as nutrient-dense foods alone are not typically associated with weight gain. We have revised the sentence to clarify that the mechanism likely relates to greater food security, increased overall caloric intake, and access to energy-dense foods, which may accompany higher household income after marriage. Additionally, we appreciate the suggestion regarding perimenopause-related physiological changes among older women.

We have added this in the revised manuscript “On the one hand, marriage may increase household income and food security, resulting in more regular meals and greater overall caloric intake, including increased access to energy-dense foods that are more strongly associated to weight gain. Among older women, age-related physiological changes including perimenopause and reduced metabolic rate may further contribute to the higher prevalence of overweight and obesity observed in married and previously married women [33].”
